# Caveolae-Mediated Transcytosis and Its Role in Neurological Disorders

**DOI:** 10.3390/biom15040456

**Published:** 2025-03-21

**Authors:** Kunjian Yang, Qian Li, Yushuang Ruan, Yuanpeng Xia, Zhi Fang

**Affiliations:** 1 Department of Neurology, Union Hospital, Tongji Medical College, Huazhong University of Science and Technology, Wuhan 430022, China; 2 Department of Rehabilitation Medicine, Tongji Hospital, Tongji Medical College, Huazhong University of Science and Technology, Wuhan 430022, China

**Keywords:** blood–brain barrier, transcytosis, caveolae, stroke, neurodegenerative disease

## Abstract

The blood–brain barrier (BBB) controls the flow of substances to maintain a homeostatic environment in the brain, which is highly regulated and crucial for the normal function of the central nervous system (CNS). Brain endothelial cells (bECs), which are directly exposed to blood, play the most important role in maintaining the integrity of the BBB. Unlike endothelial cells in other tissues, bECs have two unique features: specialized endothelial tight junctions and actively suppressed transcellular vesicle trafficking (transcytosis). These features help to maintain the relatively low permeability of the CNS barrier. In addition to the predominant role of tight junctions in the BBB, caveolae-mediated adsorptive transcytosis has attracted much interest in recent years. The active suppression of transcytosis is dynamically regulated during development and in response to diseases. Altered caveolae-mediated transcytosis of bECs has been reported in several neurological diseases, but the understanding of this process in bECs is limited. Here, we review the process of caveolae-mediated transcytosis based on previous studies and discuss its function in the breakdown of the BBB in neurological disorders.

## 1. Introduction

The blood–brain barrier (BBB) is the interface separating the central neural tissue from circulating blood, both physically and metabolically. A single layer of brain microvascular endothelial cells (bECs) is the core anatomical element of the BBB, which is covered by a basement membrane, pericytes, and astrocyte endfeet. All these cells comprise the structure of the neurovascular unit (NVU). Since the precise anatomical site of the BBB was identified about 50 years ago [1], the tight junctions between the bECs have garnered significant attention. The prevailing view in the field is that specialized tight junctions play a dominant role in the maintenance of BBB properties [2,3]. However, in recent years, new findings have demonstrated that the active suppression of transcytosis, which is associated with BBB function, is dynamically regulated during development and in response to diseases [4,5,6].

Transcytosis is the transcellular transportation of molecules via vesicles, allowing macromolecules to pass between the circulation and the interstitium. Transcytosis involves three steps: endocytosis on one side of the cell, intracellular vesicle trafficking, and exocytosis on the other side of the cell. However, the types of vesicles and different transcytosis mechanisms involved remain poorly understood. The current consensus postulates that transcytosis in the endothelial cells of the central nervous system (CNS) can be divided into two categories: receptor-mediated transcytosis (RMT), where ligand–receptor binding initiates endocytosis, and non-selective adsorptive transcytosis, where charged interactions between the molecules and the plasma membrane facilitate cargo entry [7]. There are three kinds of endocytosis in bECs: clathrin-mediated, caveolae-mediated, and micropinocytosis [8]. Micropinocytosis is an actin-dependent but coat- and dynamin-independent endocytic uptake process that generates large intracellular vesicles (macropinosomes) containing a non-selective sampling of extracellular fluid [9]. Research has demonstrated that micropinocytosis is mainly involved in the pathogens’ invasion and aging in the CNS [10,11,12,13,14]. However, clathrin-mediated and caveolae-mediated endocytosis are the two major endocytic pathways for macromolecules in bECs. Clathrin-mediated transcytosis involves the endocytosis of cargo through clathrin-coated vesicles and is ubiquitous in all cell types [15]. Most RMT is clathrin-mediated, such as transferrin and insulin transport [16]. Caveolae are flask-like vesicles of the plasma membrane that are rich in cholesterol and glycosphingolipids [17]. They were found to be major carriers of fluid and solutes across ECs. Macromolecular substances, such as albumin, are transported via caveolae-mediated transcytosis [8]. Additionally, caveolae-deficient ECs show deficiencies in the uptake and transport of albumin in the periphery [18] and the CNS [19]. Altered caveolae-mediated transcytosis of brain endothelial cells has been found to play a critical role in several neurological diseases, such as stroke, multiple sclerosis, and neurodegenerative diseases [20,21,22].

The current understanding of caveolae-mediate transcytosis is limited due to its complexity and the highly variable results obtained across in vitro models in different studies. Here, we review the process of caveolae-mediated transcytosis based on previous studies, discuss its function in the breakdown of the BBB in neurological disorders, and highlight the critical questions that remain.

## 2. Caveolae-Mediated Transcytosis

Caveolae, discovered by E. Yamada in 1955, are 50–100 nm flask-like vesicles [23] that mainly consist of proteins and lipids. Their biogenesis relies on caveolins and cavins. Caveolins are integral membrane proteins that bind to cytosolic cavins and drive caveolae vesicle formation [24]. The formation of caveolae is a prerequisite for caveolae-mediated transcytosis, which is subsequently followed by vesicle trafficking and exocytosis. These steps constitute caveolae-mediated transcytosis.

### 2.1. Endocytosis

Caveolae formation occurs at the beginning of endocytosis, which is mainly initiated by caveolin translation and then requires a complex formation process. It relies on interactions between lipids and proteins, as well as lipid–lipid and protein–protein associations [25]. Firstly, monomeric Cav-1 (8S-Cav) is inserted into the endoplasmic reticulum (ER) membrane during the translation process, which relies on the involvement of signal recognition particles [26]. Then, Cav-1 is transported to the Golgi apparatus (GA) via the coat protein complex II (COPII)-dependent pathway [27], which subsequently initiates lipid sorting. Cav-1 indirectly induces the accumulation of cholesterol around it, thus regulating the morphology of cholesterol-rich membranes [28]. Cholesterol spreads to the region of maximum negative curvature due to its intrinsic negative curvature. Phosphatidylinositol 4,5-bisphosphate (PtdIns(4,5)P2, PIP2) is directly recruited to the cholesterol-rich region, since the caveolin scaffolding domain (CSD) of Cav-1 copolymerizes with cholesterol and PIP2. While the exact mechanism of Cav-1 in the recruitment of phosphatidylserine (Ptdser, PS) remains unclear, several studies have suggested that there is a direct interaction between Cav-1 and PS [29]. The enrichment of PS and PIP2 provides charge interactions for the binding of cavin complexes. Two different lipid-binding sites in cavin-1 contribute to the selective lipid sorting of caveolae [30]. That is, PS plays a bridging role in the combination of Cav-1 and cavins. Cavins initially trimerize via the HR1 domain and then assemble into cytoplasmic oligomers. These oligomers bind to negatively charged lipid membranes and form a stable relationship with the plasma membrane in the presence of caveolins. Subsequently, the recruitment of cavins further increases the local concentration of negatively charged lipids [31]. This process facilitates the nucleation of membrane curvature, leading to the formation of unique cave pits, which likely represents a positive feedback mechanism. The binding of cavins to the CSD of Cav-1 further stabilizes the curvature. Essentially, spontaneous curvature results from the interplay between differential stress and bending modulus in lipid membranes exhibiting asymmetric lipid distributions [32]. Liquid–liquid phase separation (LLPS), driven by weak electrostatic interactions and interactions with membrane lipids, collectively facilitates the formation of plasma membrane caveolae [33].

However, this process does not continue indefinitely. The cluster of PIP2 recruits caveolae neck proteins, with EH domain-containing protein 2 (EDH2) specifically binding to the infundibular region of the caveolae when activated by ATP. EDH2 forms a ring-like structure at the neck of the caveolae. It recruits protein kinase C and casein kinase substrate in neurons 2 (Pacsin2) in the early stages and EH domain-binding protein 1 (EHBP1) in the later stages through its EH domain, potentially correlating with the membrane curvature [34]. Both Pacsin2 and EHBP1 bind to cytoskeletal actin to stabilize the caveolae in the subplasma cortical region. Receptor tyrosine kinase-like orphan receptor 1 (ROR1), as a scaffold protein for cavin-1 and Cav-1, interacts with cavin-1 via its abundant C-terminal serine/threonine domain. Two-thirds of the kinase domains bind to cavin-1, facilitating the interaction between Cav-1 and cavin-1 on the plasma membrane [35]. The expression of Cav-1 is preserved by inhibiting lysosome-dependent degradation and the associated vesicle formation. The above factors promote the stability of caveolae on the membrane and better implement their function in information transmission.

**Figure 1:**
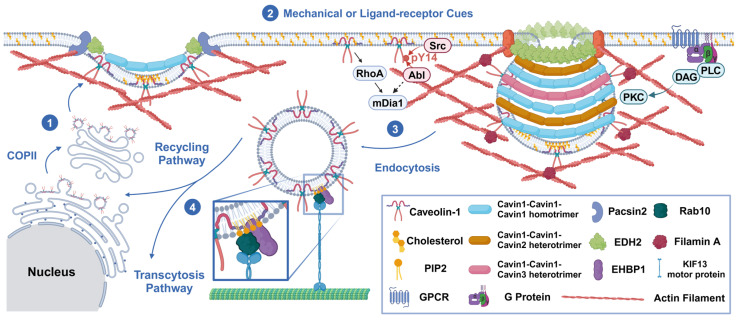
Mechanisms of the endocytosis process. (1) In a static state, caveolin-1 integrates into the plasma membrane (PM) in a COPII-independent manner, leading to lipid sorting, cavin recruitment, and a specific curvature formation of the membrane. (2) Due to mechanical tension from the extracellular matrix (ECM), integral membrane proteins, or ligands released into the circulation, the plasma membrane invaginates and forms caveolae. This process is regulated by kinases (Src, PKC, and Abl) and the depolymerization of the cytoskeleton (Abl/mDia1 and FLNa). Pacsin2, EDH2, and EHBP1 stabilize the neck of the caveolae, preventing their invagination. (3) Endocytic vesicles are transported by microtubules and dynein/kinesin (e.g., KIF13 motor protein). (4) These vesicles are then sorted into either the recycling pathway (e.g., caveolins and receptors) or the secretory pathway (e.g., cargo of transcytosis). The figure was created with BioRender (https://biorender.com/).

However, the exact triggers for the invagination of caveolae are not clear. We suggest that this biological process results from the interplay between driving forces and stabilizing factors. When Src and protein kinase C (PKC) are induced by various signal transduction pathways, they can trigger the invagination of caveolae. There are multiple sites on Cav-1 that bind to Src. The scaffold domain and palmitoylation of Cav-1 at cysteine site 156 [36] contribute to Src coupling, which leads to Cav-1 phosphorylation on tyrosine residue 14. This promotes cave swelling and the release of caveolae by causing the separation or diffusion of adjacent negatively charged N-terminal phosphotyrosine residues [37]. Src also enhances the binding of filamin A (FLNa) to Cav-1 in endothelial cells, which is associated with the phosphorylation of Cav-1. This promotes the internalization and transport of caveolae [38]. PKC acts on the proteins at the neck of the caveolae and induces instability in proteins such as EDH2, Pacsin2, and EHBP1. It phosphorylates Pacsin2 at serine 313, thereby decreasing its membrane-binding ability [39]. It also induces adaptive regulation of the cytoskeleton. PKCα mediates the phosphorylation of FLNa [40] on serine 2152, which induces the invagination of caveolae and the formation of Rab11-positive recycling endosomes. Meanwhile, Abl tyrosine kinase and mammalian diaphanous 1 (mDIA1) coordinate the co-arrangement of caveolae and actin stress fibers [41]. Subsequently, β1-integrin, integrin-linked kinase (ILK) [42], IQ motif-containing GTPase-activating protein 1 (IQGAP1), and mDIA1 [43] mediate microtubule stabilization, providing cytoskeletal support for vesicle transport. Additionally, ATP hydrolysis may lead to the depolymerization of EDH2 from the neck of the caveolae, thus mediating the endocytosis of the caveolae [44]. Subsequently, EHBP1 binds with Rab10, guiding the invaginated caveolae to the KIF13 motor protein, which initiates microtubule-mediated vesicle trafficking [45] (Figure 1).

Based on the above discussion, it is important to highlight that there are different pools of caveolae that perform distinct functions [46]. Two of the most important pools are those involved in balancing the requirements for signaling transduction (active pools for endocytosis or transcytosis) [47] and mechanosensation (static pools anchored on the membrane) [17]. This illustrates the various dynamics and functions of caveolae. This hypothesis still requires more evidence, but provides insights into the organization of caveolae. Overall, the formation process and quantity of caveolae are regulated by both membrane homeostasis and cellular signal transduction.

### 2.2. Vesicle Trafficking

The complete vesicle trafficking process initiated by caveolae-mediated endocytosis in bECs is not well understood. It is unclear whether caveolae are endocytic transport vehicles or if they indirectly induce transcytosis through endosomes. Furthermore, it appears that the trafficking pathways of endocytosis and transcytosis are different. However, due to the limitations of experimental techniques and the lack of specific cargo, it may be difficult to distinguish which pathways the vesicles follow in cells and the molecules that regulate these processes.

At present, the main hypothesis is that caveolae are regulated by Rab GTPases for transport, together with intracellular vesicles, after endocytosis [48]. The transcellular transport system consists of Rab GTPases, cytoskeleton-based pathways for the translocation of endocytic vesicles, cytoskeleton-associated motor proteins, and cargo-carrying vesicles [49]. It is currently accepted that Rab5 regulates the transport of vesicles from the plasma membrane to early endosomes (EEs) and promotes the fusion of nascent endocytic vesicles with early endosomes, as well as between early endosomes [50]. Therefore, Rab5-positive endosomes are considered to be markers of early endosomes. These Rab5-positive sorting endosomes then mature into late Rab7-positive endosomes (late endosomes, LEs) [51]. Cargo not targeted for Rab4- and Rab11-mediated recycling pathways remain in the LEs. In the last stage of endocytosis, LEs eventually mature into lysosomes or fuse with lysosomes [52]. However, in the transcytosis process, to avoid the degradation of cargo by lysosomes, sorting begins mainly in the EEs and leads vesicles to the secretory pathway [53].

Yet, Shikanai et al. [54] have reported that in cortical neurons, Rab5 and Rab21 mediate distinct EEs. The inhibition of Rab21, but not Rab5, results in decreased plasma membrane localization and total concentration of Cav-1. That is to say, Rab21 preferentially collocates with Cav-1 and regulates the caveolin-mediated endocytosis pathway, while Rab5 regulates the clathrin-mediated pathway. However, it remains to be determined whether this difference also occurs in bECs, which requires further research and experimental validation. Moreover, biomarkers, such as different types of Rab GTPases and endosomal sorting complex for promoting exit 1 (ESCPE-1) [55], as well as the secretory pathway guidance, the secretion process of cargo, and regulatory molecules still require further exploration.

The mechanisms by which Rab GTPases mediate vesicle sorting and fusion have already been defined. Membrane-bound GTP-Rab recruits Rab effectors (tethering complexes), facilitating membrane tethering and fusion. Once a vesicle is tethered to its target membrane by a Rab effector, the two lipid bilayers are brought into close proximity. The vesicle-anchored soluble N-ethylmaleimide-sensitive factor attachment protein receptors (v-SNAREs) on vesicles and target membrane-associated SNAREs (t-SNAREs) on target membrane proteins interact to form a four-α-helical bundle. These intertwined SNARE complexes catalyze membrane fusion, starting with the outer lipid bilayers and then the inner bilayers. After fusion, the cytosolic proteins N-ethylmaleimide-sensitive factor (NSF) and soluble NSF attachment proteins (SNAPs) utilize ATP to disassemble the SNARE complex, resetting it for subsequent rounds of fusion [56].

Transcytosis can generally be divided into two types based on the pathways involved. One type is receptor-mediated transport through the classical route, which includes endocytosis, endosome transport, delivery to the basolateral membrane, and subsequent secretion. The other type involves highly lipid-modified ligands, which integrate into the membrane and are sorted into LEs and multivesicular bodies (MVBs) via EEs. MVBs incorporate cargo into intraluminal vesicles and target them to the plasma membrane, where ligand-associated intraluminal vesicles are released as extracellular vesicles or exosomes [57]. Liu et al. [58] substantiated the sorting mechanism of MVBs. Myotubularin 1 (MTM1) and phosphatidylinositol 4-kinase IIα (PI4KIIα) on the surface of MVEs catalyze the stepwise transformation of phosphatidylinositol-3-phosphate (PI3P) into phosphatidylinositol-4-phosphate (PI4P), promoting the assembly of exosome complexes. This process facilitates the extracellular release of MVBs, ultimately resulting in exosome secretion. If PI4P synthesis or extracellular vesicle functionality is disrupted, the release of programmed death ligand 1 (PD-L1) via exosomes is inhibited, which subsequently causes PD-L1 accumulation and cargo degradation within lysosomes.

Microtubules and motor proteins contribute to the movement of vesicles between various compartments [59]. During endocytosis, actin motor myosin VI and adaptors, such as disabled adaptor protein 2 (Dab2) or G-α interacting protein (GAIP)-interacting protein C terminus (GIPC), bind to endosomal cargo, driving the movement of endosomes through the actin cortex [60]. Subsequently, cargo adaptors (including Hook1, Hook3, Rab45, and calcium release-activated channel regulator 2A (CRACR2A)) and dynein–dynactin complexes interact with endosomes, driving EEs toward the minus ends of microtubules [61]. EEs associated with microtubules can simultaneously interact with plus-end-directed kinesins and minus-end-directed dyneins, which determines the direction of endosomes [62]. And microtubule-associated proteins, such as tau and microtubule-associated protein (MAP), can also enhance or inhibit plus-end-directed movements, thereby influencing the directionality of endosome transport [63]. Finally, PhosPI3P on EEs recruits Rab7, replaces Rab5, and promotes the maturation of EEs into LEs. Alternatively, it recruits the kinesin family member KIF16B, a minus-end-directed motor protein, which segregates cargo from Rab5-positive EEs and transports it to Rab11-positive recycling endosomes. This process is crucial for lysosomal fusion and proper lysosomal positioning [64]. However, the motor proteins and adaptors involved in the secretory pathway of transcytosis are not yet fully understood. Additionally, the relationship between the Rab11-mediated recycling pathway and the secretory pathway remains unclear. Moreover, due to the non-selectivity of cargo transported by caveolae, it is not yet clear whether caveolae vesicles have special motor proteins and adaptor transport.

Lastly, in trafficking pathways, caveosomes are controversial organelles. When studying the intracellular trafficking of simian virus 40 (SV40), Lucas Pelkmans identified an organelle containing Cav-1 but lacking markers of classical endocytic or exocytic pathways [65]. Notably, this organelle exhibited a non-acidic pH. Raluca G. suggested that caveosomes are distinct organelles that do not originate from the maturation of caveolae [66]. However, Robert G. Parton believes that they are essentially LEs [67].

### 2.3. Exocytosis

The last process in transcytosis is exocytosis, which transfers the material to the basal–lateral membrane. This process has been well studied in synapses [68] and astrocytes [69] but is relatively lacking in the research on bECs. Classic exocytosis can be divided into a regulated secretory pathway and a constitutive secretory pathway. However, it is unclear whether the exocytosis process in transcytosis follows these pathways and whether its mechanisms vary depending on the specific substances being transported. Although there is no exact description of the exocytosis process of transcytosis in endothelial cells, this mechanism is considered to be conserved in different cell types.

**Figure 2:**
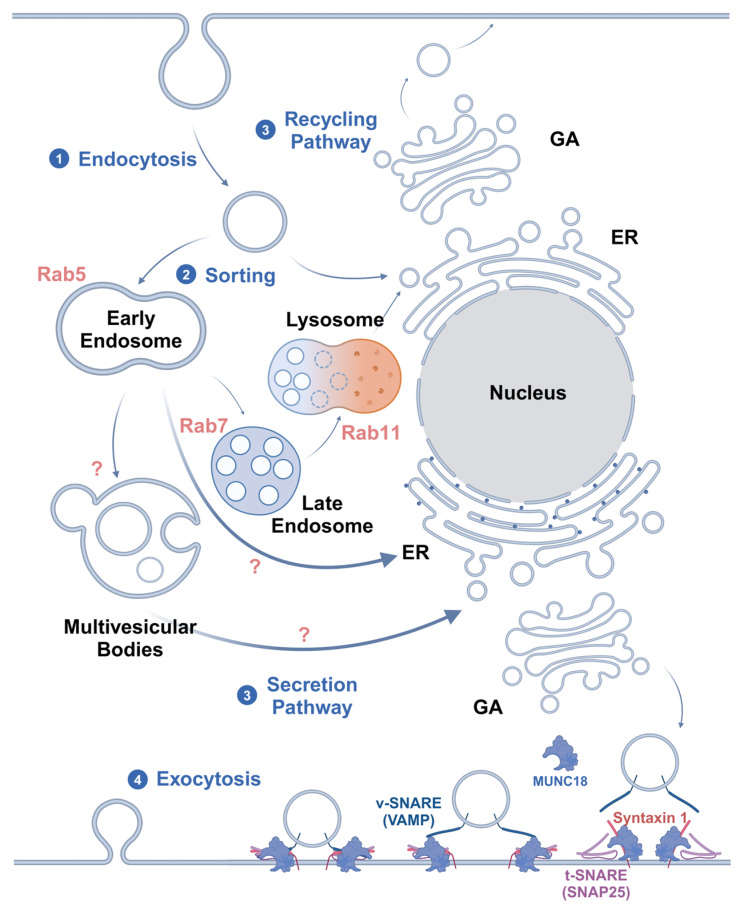
A summary of the mechanisms of endosome sorting and exocytosis. After endocytosis, (1) Rab5 endocytic vesicles are transported to EEs for sorting. (2) The fusion process of vesicle trafficking is implemented in a SNARE-mediated manner. (3) For nutrient degradation and membrane protein recycling, Rab7 directs the vesicles into LEs, which then fuse with lysosomes. Meanwhile, the vesicles of transcytosis that transport highly lipid-modified ligands are sorted into MVBs and then directed to the plasma membrane for secretion through the ER and GA. Other ligands, such as soluble ones, may be directly sorted into the secretory pathway from EEs. However, the sorting signals for this process are not clear. (4) Eventually, exocytosis is initiated by Munc18 in a SNARE-dependent manner, transporting cargo across the endothelial cells. The figure was created with BioRender.

The current consensus is that SNAREs may be involved in the exocytosis of basolateral vesicles. This process is Ca^2+^-dependent. Initially, the Sec1/Munc18-like (SM) protein Munc18-1 binds to Syntaxin1, changing its formation from closed to open. Then, VAMP2 on the vesicles can form a tight complex with SNAP-25. At this time, Munc18-1 remains bound to Syntaxin1 within the assembled SNARE complex, but it switches its binding mode to interact with the SNAREs, assembling the trans-SNARE complex. Thereafter, complexin binds the complex to further enhance secretory vesicle initiation. The change in the formation of this SNARE/SM complex prepares it for Ca^2+^ activation. Subsequently, the fusion pore opens, and the vesicle membrane fuses with the cell membrane, releasing the cargo and integrating the receptors into the plasma membrane. Simultaneously, the trans-SNARE complex is converted into the cis-SNARE complex. Afterward, NSF and SNAPs hydrolyze ATP and contribute to the recycling of SNAREs [70] (Figure 2).

Due to its complexity, caveolae-mediated transcytosis—especially the process of vesicle trafficking—is not fully understood. More investigations need to be carried out to further elucidate the mechanisms behind it.

Caveolae-mediated transcytosis in bECs is suppressed under normal conditions, which helps maintain the function of the BBB and is essential for the homeostasis of the CNS and the normal function of neurons. Several factors are involved in maintaining low-rate transcytosis. Pericytes surround bECs at a precise ratio and facilitate extensive signaling in them. Studies have revealed that both pericyte-deficient mice, generated by manipulating a signaling pathway that normally recruits pericytes [71,72,73], and pericyte density-increased mice, generated by depleting Foxf2 [74], showed leaky barriers. In both models, leakage was caused by increased transcytosis; however, it is not known whether it was mediated by caveolae.

The lipid composition of bECs is different from that of the peripheral endothelium. The major facilitator superfamily domain-containing 2a (Mfsd2a) is specifically expressed in bECs and suppresses the number and rate of caveolae-mediated transcytosis [75]. Andreone et al. [76] observed a significant increase in the number of endocytic vesicles and the leakage of the BBB in Mfsd2a^−/−^ mice, but the tight junctions between the bECs remained intact. Additionally, Wang et al. [77] directly upregulated the transcription of Mfsd2a via wnt/β-catenin/TCF signaling, thereby suppressing transcytosis and maintaining the integrity of the BBB. Mfsd2a mediates the transport of omega-3 fatty acid docosahexaenoic acid (DHA), leading to changes in the lipid composition and a decrease in the concentration of lipids involved in caveolae formation [76]. Previous studies have shown that Mfsd2a interacts with Spinster Homolog 2 (SPNS2) to export S1P across brain ECs, thereby regulating the formation and maintenance of the BBB [78].Intracellular signaling molecules also mediate the abundance of Mfsd2a. bEC-specific phosphatase and tension homolog (Pten) inhibits the AKT/E3 ubiquitin ligase NEDD4-2-mediated degradation of Mfsd2a, thereby maintaining the integrity of the BBB [79].

The variation in caveolae-mediated transcytosis can lead to BBB breakdown, which plays an important role in neurological disorders.

## 3. Caveolae-Mediated Transcytosis in Neurological Disorders

### 3.1. Ischemic Stroke

It is well known that the integrity of the BBB is disrupted after an ischemic stroke. The prevailing view in the field is that the destruction of intracellular tight junctions (TJs) is the main reason for BBB breakdown. However, with research on transcytosis deepening, recent studies using electron microscopy have shown that increased transcytosis is the primary culprit [5,80,81,82,83,84]. Knowland et al. [5] suggested a two-phase stepwise breakdown model in which caveolae-mediated transcytosis plays a decisive role in the early phase (6–12 h) of BBB leakage, while TJ disruption contributes mainly in the later phase (24–48 h). Nahirney et al. [83] demonstrated that BBB permeability was associated with a striking increase in endothelial caveolae and vacuoles at early and delayed changes (3 versus 72 h) in both young and aged mice (3–4 versus 18 months). Endothelial vesicles and transcytosis-related proteins, such as Cav-1, were observed to be upregulated by hyperglycemia following ischemia and reperfusion [80]. Ultrastructural examination of the vessels along the hippocampal fissure revealed that the endothelial cytoplasm contained horseradish peroxidase (HRP)-filled vesicles or vacuoles in close proximity to the basal lamina, which appeared to be slightly electron-dense in ischemic reperfusion [81]. The endothelium exhibited enhanced transendothelial vesicle trafficking and signs of degeneration at 5 and 25 h after ischemia induction [82].

The endothelial glycocalyx is a layer of membrane-associated proteoglycans and glycoproteins that line the luminal surface of the endothelium. A negatively charged glycocalyx facilitates the crossing of positively charged proteins in the vascular lumen through the BBB via caveolae-mediated transcytosis driven by electrostatic interactions [85]. Under ischemic and hypoxic conditions (transient middle cerebral artery occlusion (t-MACO) model), the glycocalyx (GCX) undergoes enzymatic degradation, facilitating the interaction between syndecan-1 and Src by enhancing the binding of phosphorylated syndecan-1 to the Src SH2 domain. This process leads to the rapid regulation of cytoskeletal proteins, promoting caveola-mediated endocytosis [86]. This effect has also been observed in BBB breakdown caused by cardiac arrest and status epilepticus [87]. Glial growth factor 2 (GGF2), a recombinant version of neuregulin-1β that can stimulate glial cell proliferation and differentiation, has been demonstrated to dose-dependently reverse the acute ischemic stroke-induced upregulation of vesicular transcytosis and Cav-1, as well as the downregulation of Mfsd2a [88]. Storax, a natural resin from Liquidambar, has been demonstrated to significantly increase the expressions of Mfsd2a and PDGFR-β, decrease the expressions of Cav-1 and AQP4, and consequently inhibit caveolae-mediated transcytosis at the BBB in a focal stroke model of rats [89].

Although the relationship between Cav-1 and TJs is still disputed, transcytosis and TJs are not completely irrelevant. The increase in the caveolae-mediated transcytosis rate may lead to the disruption and remodeling of TJs, such as ZO-1 and Claudin5 [90]. In this context, bone marrow mesenchymal stem cell-derived extracellular vesicles (BMSC-EVs) and bEC-derived EVs (bEC-EVs) play protective roles in maintaining the integrity of the BBB. Additionally, under hypobaric hypoxia, induced NRF1 upregulates Cav-1 at the transcriptional level, which facilitates the endocytosis of Claudin5, leading to its autophagic degradation [91]. ROS can also induce this effect.

Notably, although Cav-1 is a key component of caveolae, beyond its role in facilitating caveolae-mediated transcytosis, it regulates various signaling pathways via its CSD domain. These pathways are associated with cellular metabolism, autophagy and oxidative stress, senescence, cholesterol trafficking, vascular tone, synapse transmission, neural inflammatory responses, and more [92]. Additionally, Cav-1 can also interact with various receptors to regulate their downstream signaling pathways, including receptor tyrosine kinases (RTKs) [93] and G-protein-coupled receptors (GPCRs) [94]. Given the complexity of Cav-1’s functions, understanding the specific mechanisms through which it exerts its effects is significant. This complexity also underpins its multifaceted role in neuroinflammation after acute ischemia stroke (IS). Meanwhile, neuroinflammation after IS involves the participation of various cells and molecules, with highly complex mechanisms. For further details, please refer to another review [95], as these topics are not elaborated upon here.

A study demonstrated that a low serum Cav-1 level predicted symptomatic hemorrhage following r-tPA treatment in acute IS patients [96]. In mice, rt-PA treatment administered 4 h after MCAO upregulated ischemia-induced VEGF expression and VEGFR-2 phosphorylation in an LDL receptor-dependent manner, leading to increased endothelial cell caveolae-mediated transcytosis [97]. The use of anti-Nogo-A antibodies can partially reverse VEGF-induced BBB leakage [98]. However, it remains unclear whether this effect is related to VEGF-enhanced transcytosis. Defects in the Nogo-B receptor also lead to BBB disruption, but this effect is associated with TJs [99]. Filchenko et al. [100] found that Cav-1 deficiency led to reduced AQP4 expression and impaired perivascular AQP4 coverage following cerebral ischemia. This was associated with morphological changes in reactive astrocytes and exacerbated brain swelling. However, the details of the regulatory mechanism remain unknown.

### 3.2. Other Cerebrovascular Injury

Mfsd2a is a critical factor for BBB maintenance via caveolae-mediated transcytosis, as previously described. A study demonstrated that Mfsd2a attenuated intracerebral hemorrhage-induced BBB disruption by inhibiting vesicular transcytosis, likely via trafficking-related proteins (Srgap2, Stx7, and Sec22b) [101]. In a model of arachnoid hemorrhage, Mfsd2a showed a similar effect. When the supply of omega-3 fatty acids was halted, Mfsd2a lost its ability to inhibit caveolae and protect the BBB [102].

Under conditions of chronic cerebral hypoperfusion, increased endothelial transcytosis was observed in the corpus callosum (CC). This process may be regulated by TGF-β/Smad2 signaling [103]. Caveolae, particularly Cav-1, play a crucial role in the development of hypertension and the subsequent impairment of the BBB. Fragas et al. [104] observed that hypertension increased caveolae-mediated transcytosis in the paraventricular nucleus (PVN) of the hypothalamus, leading to disruption of the BBB and enhanced leakage into the brain parenchyma. However, current information on the mechanisms of BBB defects caused by hypertension is scarce and contradictory. It is currently established that Cav-1 competitively inhibits eNOS [105], but this is not the sole mechanism responsible for the pathogenesis of hypertension or the primary cause of BBB damage.

Due to its lipid transport function, caveolae/Cav-1 are associated with atherosclerosis. Although LDL transcytosis across the BBB is mediated by LDL receptors, caveolae generally mediate the transcytosis of LDL and ox-LDL (accounting for 70%) via the scavenger receptors SR-B1 and activin-like kinase receptor 1 (ALK1) [106]. LDL particles are colocalized with SR-B1 in endothelial cell intracellular vesicles in vivo, and LDL transcytosis across endothelial monolayers requires direct binding to SR-B1 and an 8-amino acid cytoplasmic domain of the receptor, which recruits the guanine nucleotide exchange factor dedicator of cytokinesis 4 (DOCK4). SR-B1 drives endothelial cell LDL transcytosis via DOCK4 to promote atherosclerosis [107]. Once LDL enters the endothelial cells, it undergoes modification, which activates endothelial cells and recruits monocytes, accelerating the formation of fatty streaks, a core mechanism in atherosclerosis. In addition to LDL transcytosis, caveolae may also mediate the transport of HDL across endothelial cells and regulate reverse cholesterol transport [108]. The absence of caveolae weakens LDL infiltration and reduces the endothelial cell activation induced by TNFα and IL1β, significantly altering the composition of the subendothelial basement membrane [109]. Caveolae can regulate the endocytosis, degradation, and secretion of fibronectin via exosomes, thereby reducing fibronectin accumulation [110]. The deposition of fibronectin in the intima of areas prone to atherosclerosis alters integrin signaling, promoting endothelial cell inflammation and atherosclerosis [111]. Additionally, Cav-1 deficiency weakens caveolae-mediated leukocyte migration, hindering their infiltration into the arterial wall and slowing the progression of atherosclerosis [112]. Therefore, caveolae-mediated transcytosis is a significant factor in the pathogenesis of cerebrovascular atherosclerosis. Moreover, caveolae provide a platform for the inflammatory response in atherosclerosis and reverse cholesterol transport, involving multiple receptors and signaling pathways. The details of these mechanisms are discussed in another review [113].

### 3.3. Caveolae-Mediated BBB Breakdown and Neurodegeneration

#### 3.3.1. Alzheimer’s Disease (AD)

At present, the pathogenesis of Alzheimer’s disease (AD) remains unclear. The predominant hypothesis involves a beta-amyloid (Aβ)-induced pathological cascade: Aβ deposition induces tau hyperphosphorylation and tangle formation, followed by neuronal death. The upstream mechanisms of Aβ deposition are not well understood, but the normal functioning of the NVU in maintaining cerebrovascular homeostasis is critical for preserving neuronal function and clearing harmful metabolic byproducts [114]. This also explains why cerebrovascular diseases increase the risk of developing AD.

The National Alzheimer’s Coordinating Centre evaluates the contribution of cerebrovascular diseases and vascular risk factors to the development of AD [115]. Among 4629 AD patients, 79.9% exhibited vascular pathology, including cerebral amyloid angiopathy (40.8%), atherosclerosis (39.8%), arteriolosclerosis (34.6%), cerebrovascular disease (32.3%), multiple microinfarcts (20.1%), lacunes (19.9%), large infarcts (12.7%), and arteriosclerotic leukoencephalopathy (9.3%). This suggests a strong association between AD and cerebrovascular injury, as well as BBB disruption. The dual-hit hypothesis of AD states that the initial hit causes damage to the cerebral microcirculation, disrupts the BBB, and increases its permeability, allowing neurotoxic blood-derived molecules and peripheral inflammatory cells to infiltrate the CNS [116]. This damages neurons and triggers neuroinflammation. Simultaneously, reduced cerebral blood flow (CBF) and inadequate brain perfusion exacerbate ischemic and hypoxic changes. The disruption of vascular homeostasis contributes to increased Aβ deposition and impaired clearance in the brain parenchyma, leading to associated neurotoxicity and cognitive dysfunction. Throughout this process, caveolae-mediated transcytosis plays a vital role in BBB breakdown in cerebrovascular disease and the dysfunction of Aβ clearance. Wang et al. [117] found that caveolae mediate Aβ42 transcytosis through bECs, while clathrins mediate Aβ40. Additionally, the Aβ42/Aβ40 ratio could predict amyloid-PET status, indicating the pathological severity. However, more evidence must be found to support this hypothesis. A prospective cohort study of 74,754 individuals that was focused on transcytosis genes and the risk of dementia and stroke suggested that clathrin-mediated endocytosis in the clearance of amyloid-β across the BBB is important for the integrity of both brain tissue and cerebral vessels [118].

Neurodegeneration resulting from neuroinflammation is also a significant mechanism that leads to AD and permanent cognitive deficits [119]. In the early phase of neuroinflammation, neutrophils and T cells, particularly CD4^+^ T cells, recognize adhesion molecules, such as intercellular adhesion molecule-1 (ICAM-1) and vascular cell adhesion molecule-1 (VCAM-1) [120]. This initiates the caveolae-mediated transcytosis process. Subsequently, matrix metalloproteinase 2 (MMP2) and MMP9, secreted by infiltrating neutrophils, lead to the degradation of the extracellular matrix and the basal membrane [121], facilitating further infiltration of inflammatory cells. At the same time, astrocytes and microglia are activated and secrete inflammatory cytokines. MMP2/9 and cytokines then degrade TJs, including occludin and claudin-5, contributing to the late phase of BBB breakdown. This opens the paracellular pathway, allowing more immune cells (Th1 and Th17 cells) to cross the barrier [122]. IL and TNF also recruit more neutrophils and T cells to enhance the inflammatory reaction, thus inducing neural damage and cognitive deficits [123].

Aging is the primary risk factor for many neurodegenerative diseases, including AD [124]. The loss of BBB integrity, particularly the diminished ability to inhibit caveolae-mediated transcytosis, is a hallmark of natural aging. In aged mice, acidic sphingomyelinase (ASM) is upregulated and regulates caveolae–cytoskeleton interactions through the protein phosphatase 1-mediated dephosphorylation of ezrin/radixin/moesin (ERM) proteins and apoptosis. This process increases the quantity and rate of caveolae-mediated transmembrane transport, leading to BBB leakage and accelerating the progression of neurodegenerative disorders [125]. This further validates that the dysfunction of the BBB is highly associated with the generation of neurodegenerative diseases, which induce chronic neuroinflammation.

Agonist-induced caveolae-mediated GLP-1 invagination and activation can stimulate PKA/AMPA/ULK1-modifying autophagy/mitophagy, which contributes to neuroprotection and neurogenesis [126]. Furthermore, GLP-1 may affect amyloid-beta peptide aggregation in AD through the phosphoinositide-3 kinase/AKT/mTOR signaling pathway [127]. The function of GLP-1 is partially related to the NMDA receptor. The NMDA receptor is a crucial ionotropic glutamate receptor involved in processes such as brain development, learning, and memory. It plays a key role in synaptic plasticity and neuronal protection [128]. However, the mechanisms underlying its functions remain poorly understood, which may be one of the reasons for the current suboptimal therapeutic outcomes.

Cav-1 dysregulation may affect synaptic integrity [129], accelerating disease progression due to its regulatory effect on cell signaling transduction [130]. However, this is not associated with the transport process mediated by caveolae. The main role of caveolae-mediated transcytosis is to regulate BBB integrity to protect neurons from peripheral inflammatory factors and circulatory toxins.

#### 3.3.2. Other Neurodegenerative Diseases

Parkinson’s disease (PD) is a motor-deficient neurodegenerative disease caused by neuronal death in the substantia nigra (SN). Its pathological features are Lewy bodies (the abnormal accumulation of α-synuclein and protein inclusions in neurons). Disruption of the BBB is observed in the development of PD, but the underlying mechanism remains unclear. M. Ohlin et al. [131] reported that long-term levodopa treatment induced the expression of vascular endothelial growth factor (VEGF) in the basal ganglia nuclei in a dose-dependent manner, leading to angiogenesis and basal ganglia leakage. This process is closely associated with the insufficient inhibition of caveolae-mediated transcytosis during the early stages of vascular formation. During vascular development, caveolae-mediated transcytosis is progressively suppressed, which contributes to the establishment of the BBB [132]. Astrocytes play a critical role in maintaining the BBB by regulating Cav-1 expression at the NVU through the Wnt/β-catenin pathway. This regulation suppresses vesicle abundance, thereby maintaining a low rate of transcytosis, which is essential for maintaining the integrity of the BBB [133]. Furthermore, caveolae (Cav-1 Tyr14-phosphorylation) may also mediate the spread of abnormal α-synucleins [134]. However, the relationship between BBB leakage and PD development is under-investigated. Additionally, whether the abnormal clearance rate of α-synuclein is associated with caveolae requires further validation.

Huntington’s disease (HD) is directly associated with caveolae-dependent cholesterol transport defects [135]. The abnormal huntingtin protein inhibits caveolae-associated post-Golgi transport from the endoplasmic reticulum and Golgi apparatus to the plasma membrane, resulting in intracellular cholesterol accumulation. This accumulation suppresses cholesterol synthesis and disrupts lipid homeostasis, which may lead to cytotoxicity. However, the precise mechanisms underlying this effect remain unclear.

Similar to the BBB, Winkler et al. [136] found evidence of blood–spinal cord barrier (BSCB) breakdown and a reduction in pericytes in amyotrophic lateral sclerosis. The decreased inhibition of pericytes to caveolae-mediated transcytosis may be the mechanism behind BSCB breakdown. It is clear that dysregulation of caveolae function may exacerbate ALS, where synaptic signaling pathways are crucial for motor neuron survival [137]. However, there is no explicit evidence showing whether caveolae-mediated transcytosis engages in BSCB breakdown or not. Furthermore, Winkler et al. [138] suggested that BSCB breakdown contributes to early motor neuron degeneration in amyotrophic lateral sclerosis (ALS) mice. However, Waters et al. [139] argued that BSCB damage does not correlate with aggregations of phosphorylated TAR DNA-binding protein 43 (TDP-43), which marks the loss of lower motor neurons. This suggests that BSCB leakage and TDP-43 pathology represent distinct and unrelated pathological processes in ALS. Thus, the role of BSCB disruption in the pathogenesis and progression of ALS remains unclear, and the factors that lead to BSCB disruption and their underlying mechanisms require further investigation.

### 3.4. Autoimmune Neuroinflammation and Infection

Autoimmune neuroinflammation is a group of disorders accompanied by neuroinflammation, functional deficits, and motor dysfunction [140]. Although the exact pathogenesis of multiple sclerosis (MS) remains unclear, its pathological process can be broadly summarized into three main phases: abnormal peripheral immune activation of autoreactive responses, pathogenic immune infiltration targeting the CNS, and subsequent demyelination and motor dysfunction [141]. Therefore, understanding how peripheral immune cells and cytokines cross the BBB is essential for understanding MS pathogenesis. Caveolae-mediated transcytosis could transport Th1 cells across the bECs, thus contributing to the inflammatory effects [21]. Additionally, Cav-1 regulates the expression of ICAM-1 and VCAM-1, which mediates the adhesion of Th1 cells and initiates the transcytosis process [142].

Though micropinocytosis is the main way of pathogens’ invasion in the CNS, Caveolae-mediated transcytosis is also associated with pathogen infection. For example, Porphyromonas gingivalis (P. gingivalis) bacteremia significantly inhibits the expression of Mfsd2a, inducing an increased rate of caveolae-mediated transcytosis and BBB leakage. Additionally, various viruses can be transported into cells via caveolae [143]. For example, SV40, which is now used for exploring the sorting mechanism of caveolae vesicles, can be transported to the ER via caveolae [144]. The permeability of the BBB, neuroinflammation, and cognitive deficits are associated with SARS-CoV-2 infection [145]. Additionally, there are many other viruses that invade host cells through caveolae-mediated endocytosis [146]. Regulating this pathway may play an important role in combating CNS infections.

## 4. Conclusions and Future Perspectives

This review article delved into the processes and pivotal roles of caveolae-mediated transcytosis in bECs in neurological disorders. Caveolae, as unique membrane structures in bECs, are not only involved in lipid homeostasis and the migration of inflammatory cells but also play a key role in the pathophysiological processes of various neurological diseases. We provided a detailed discussion of the process of caveolae-mediated transcytosis and its role in the disruption of the BBB, particularly its impact on ischemic stroke, Alzheimer’s disease, and other neurodegenerative disorders.

The complexity of caveolae lies in their involvement in various metabolic and signal transduction pathways. Furthermore, their protein and lipid components, such as kinases, receptors, and cytoskeleton elements in the cytoplasm, modulate the formation and dynamics of caveolae, which are vital for maintaining the integrity of the BBB and regulating transcytosis.

In the context of neurological diseases, caveolae-mediated transcytosis plays a significant role in the disruption of the BBB and the development of brain edema. In acute ischemic stroke, caveolae-mediated transcytosis plays a crucial role in early BBB disruption. In contrast, in Alzheimer’s disease, caveolae may be involved in the transport and clearance of Aβ peptides, thereby affecting disease progression. Additionally, caveolae are involved in hypertension, atherosclerosis, and other neurodegenerative diseases, where their dysfunction may lead to BBB disruption and neurological damage. In autoimmune neuroinflammatory and infectious diseases, caveolae-mediated transcytosis is equally important. For instance, in multiple sclerosis, caveolae may facilitate the BBB crossing of autoreactive immune cells, exacerbating neuroinflammation and demyelinating lesions.

Despite a growing understanding of the role of caveolae in neurological diseases, there are still many unknowns that require further exploration. Future research should focus on the specific mechanisms of caveolae in the functioning of the BBB and neuroinflammation, as well as how they respond to different pathological stimuli. Moreover, the potential of caveolae as a therapeutic target in the treatment of neurological diseases also requires further assessment. For example, by modulating the function of caveolae, new therapeutic strategies may be developed to protect or repair the BBB, reduce brain edema, and slow the progression of neurodegenerative diseases.

In summary, the multifunctionality and complexity of caveolae in neurological diseases offer abundant opportunities for future research. By further elucidating the mechanisms of caveolae in diseases, we can anticipate the development of new drug delivery therapeutic strategies for the CNS to more effectively treat and prevent neurological diseases.

## Data Availability

No datasets were generated or analyzed during the current study.
